# Risk of viral respiratory infection associated with shared washroom between adjoining rooms: a test-negative study

**DOI:** 10.1017/ice.2025.10262

**Published:** 2025-10

**Authors:** Victoria Williams, Karoleen Volpentesta, Melisa Avaness, Christina Chan, Radhika Chawla, Amna Rizvi, Payton Bayley, Rob Kozak, Jerome A. Leis

**Affiliations:** 1 Sunnybrook Health Sciences Centre, Toronto, Ontario, Canada; 2 Centre for Quality Improvement and Patient Safety, University of Toronto, Toronto, Canada; 3 Temerty Faculty of Medicine, University of Toronto, Toronto, Canada

## Abstract

In test-negative study of residents exposed to viral respiratory infection (VRI), odds of VRI (excluding SARS-CoV-2) was higher with shared room (OR = 2.28, 95% CI, 1.53–3.40) and shared adjoining washroom (OR = 1.65, 95% CI, 1.03–2.64) than neighboring rooms. Measures recommended for exposed residents in shared rooms should be considered for shared washrooms.

Shared rooms compound transmission risk of viral respiratory infection (VRI) in nursing homes due to resident proximity.^1–3^ However, the role of a shared washroom between adjoining rooms (sometimes referred to as Jack and Jill rooms) has not been systematically evaluated.

Airflow patterns have been implicated in risk of transmission between adjacent rooms and closing doors can mitigate this risk.^4,5^ We hypothesized that adjoining shared washrooms may be similarly associated with an increased risk as compared to separated adjacentrooms without a shared washroom.

## Methods

At a federal nursing home for Canada’s Veterans made up of 405 beds in 264 rooms across 12 units, we conducted a test-negative study of residents with VRI between January 1, 2010, and December 31, 2023, where a cohort of exposed residents was identified before case status was known, then analyzed as a case-control study nested within the cohort. Research ethics board approval was obtained.

This facility has five possible room configurations; private room with dedicated washroom, shared room (2 or 3 beds) with an attached washroom, adjoining private rooms with shared washroom, and adjoining shared rooms (2 beds) with shared washroom. Residents with new onset of symptoms of VRI were routinely tested throughout the study period using a multiplex real-time polymerase chain reaction (RT-PCR) panel with targets for influenza A (H1, H3) and B, respiratory syncytial virus, human metapneumovirus, adenovirus, parainfluenza types 1–4, human coronaviruses (OC43, 229E, HKU1, NL63), and enterovirus/rhinovirus. SARS-CoV-2 was added in 2020.

Exposure variable was room design defined as exposure to a resident (index) with laboratory-confirmed symptomatic VRI who was a roommate (shared room), or shared a washroom between adjoining rooms (shared washroom), or residing in an adjacent room separated by a wall without sharing a washroom (neighboring room). A case was an exposed resident who developed VRI symptoms and tested positive for the same virus up to 5 days after the index resident’s positive test date. Exposed residents who did not test positive for the same VRI within 5 days after the index resident’s positive test date were designated as controls. Exposed residents who were asymptomatic, had an indeterminate test result without a subsequent confirmatory result within 5 days, or who had a laboratory-confirmed VRI with the same virus within 90 days prior to the exposure were excluded. In the event that the exposed resident had multiple concurrent exposures, only the exposure with the highest perceived risk (shared room > shared washroom > neighboring room) was included.

The primary outcome was the odds of VRI associated with different room configurations (shared room, shared washroom, and neighboring room). Secondary attack rate (SAR) was also calculated, defined as the incidence of laboratory-confirmed VRI within 5 days among exposed residents. A logistic regression model was calculated and presented as Odds Ratios (OR) with 95% confidence intervals (CI) and *p* < 0.05 considered statistically significant. This model adjusted for the following pre-specified predictors: unit type (independent, physical impairment, and cognitive impairment), respiratory virus, concurrent transmission on the unit (2 unrelated cases identified within 5 days) and receipt of the SARS-CoV-2 vaccine by the exposed resident within 6 months. Separate models were calculated for VRI excluding SARS-CoV-2 and SARS-CoV-2 only. Sensitivity analysis was performed extending the post-exposure follow-up period to 10 days.

## Results

There were 5199 total resident VRI exposures during the study period, of which 580(11.2%) were excluded due to a previous VRI with the same virus within 90 days, 282(5.4%) due to concurrent exposures, and 6(0.1%) due to the absence of VRI symptoms. Among the 4331(83.3%) eligible VRI exposures, there were 241 cases and 4090 controls, and SAR of 5.6%. Characteristics of resident exposures are presented in Table 1. Most exposed residents were male (89.0%) with a mean age of 92.2(±4.9) years. Residents were most frequently exposed to enterovirus/rhinovirus (1668/4331, 38.5%) and the SAR differed by VRI (SARS-CoV-2 = 25.7%, influenza A = 5.2%, enterovirus/rhinovirus = 5.0%, influenza B = 4.9%, human coronavirus = 3.6%, RSV = 3.2%, hMPV = 1.9%, parainfluenza = 1.7%). Concurrent transmission on the unit was common (51.1%) and differed by VRI, being more common for SARS-CoV-2 (82.3%) as opposed to other VRIs combined (48.7%) and being greater in scope, with a median cluster size of 10 cases (SARS-CoV-2) versus 2 (other VRI).

**Table 1.** Baseline characteristics of eligible resident exposures to viral respiratory infections between January 2010 and December 2023 (*n* = 4331)

	All exposures (*n* = 4331)	Cases (*n* = 241)	Controls (*n* = 4090)
Resident age (mean yrs ±SD)	92.2 ± 4.9	93.3 ± 5.0	92.2 ± 4.9
Resident sex (% male)	3856(89.0)	212(88.0)	3644(89.1)
Type of unit (%)			
Independent	1213 (28.0)	67(27.8)	1146(28.0)
Physical impairment	1669(38.5)	87(36.1)	1582(38.7)
Cognitive impairment	1449(33.5)	87(36.1)	1362(33.3)
Type of exposure (%)			
Neighboring room	1854(42.8)	78(32.4)	1776(43.4)
Shared room	1027(23.7)	78(32.4)	949(23.2)
Shared washroom	1450(33.5)	85(35.3)	1365(33.4)
VRI exposure (%)			
Human coronavirus	728(16.8)	26(10.8)	702(17.2)
RSV	314(7.3)	10(4.1)	304(7.4)
Enterovirus/rhinovirus	1668(38.5)	83(34.4)	1585(38.8)
hMPV	260(6.0)	5(2.1)	255(6.2)
Parainfluenza	414(9.6)	7(2.9)	407(10.0)
Influenza A	463(10.7)	24(10.0)	439(10.7)
Influenza B	184(4.2)	9(3.7)	175(4.3)
SARS-CoV-2	300(6.9)	77(32.0)	223(5.5)
Unit transmission (%)	2213(51.1)	215(89.2)	1998(48.9)

SD, standard deviation; VRI, viral respiratory infection; hMPV, human metapneumovirus; RSV, respiratory syncytial virus, SARS-CoV-2, severe acute respiratory syndrome coronavirus 2.

In univariate analysis, odds of VRI was higher in shared rooms (OR 1.87, 95% CI 1.35–2.59; *p* < .001) and shared adjoining washrooms (OR 1.42, 95% CI 1.03–1.94; *p* = .03) as compared to neighboring rooms.

In multivariate analysis, only shared rooms remained significant when SARS-CoV-2 was included (adjusted OR 2.06, 95% CI 1.46–2.91; *p* < .001) (Table 2, Model 1). Residing in a physical impairment unit, the presence of concurrent transmission on the unit and exposure to SARS-CoV-2 were all significantly associated with higher odds of VRI. When SARS-CoV-2 was excluded from all VRI, both shared rooms (aOR 2.28, 95% CI 1.53–3.40; *p* < .001) and shared washrooms between adjoining rooms (aOR 1.65, 95% CI 1.03–2.64, *p* = .037) remained associated with higher odds of VRI (Table 2, Model 2). Similar results were observed in sensitivity analysis of exposure follow-up period extended to 10 days (data not shown). Finally, in analysis of SARS-CoV-2 only, the odds of infection was not significantly associated with room configuration (Table 2, Model 3).

**Table 2.** Multivariate analysis of room configuration on odds of viral respiratory infection following laboratory-confirmed resident exposure within 5 days

Risk factor	Model 1 (All VRI) *n* = 4331		Model 2 (All VRI excluding SARS-CoV-2) *n* = 4031		Model 3 (SARS-CoV-2 Only) *n* = 300	
	aOR (95% CI)	*p*-value	aOR (95% CI)	*p*-value	aOR (95% CI)	*p*-value
Type of exposure						
Neighboring room (control)						
Shared room	**2.06 (1.46-2.91)**	**< .001**	**2.28 (1.53–3.40)**	**< .001**	1.58 (0.75–3.36)	.23
Shared washroom	1.39 (0.94–2.06)	.10	**1.65 (1.03–2.64)**	**.037**	0.95 (0.46–1.96)	.88
Type of unit						
Independent (control)						
Physical impairment	**1.73 (1.15–2.60)**	**.009**	**1.74 (1.06–2.85)**	**.028**	2.06 (0.96–4.45)	.065
Cognitive impairment	1.22 (0.86–1.73)	.27	1.34 (0.88–2.04)	.17	0.88 (0.45–1.72)	.70
Unit transmission	**6.78 (4.43–10.37)**	**< .001**	**5.88 (3.79–9.12)**	**< .001**	**28.87 (3.81–219.05)**	**.001**
VRI						
Human coronavirus (control)						
RSV	0.91 (0.43–1.94)	.80	0.94 (0.44–2.00)	.87		
Enterovirus/rhinovirus	1.34 (0.85–2.12)	.21	1.32 (0.83–2.09)	.24		
hMPV	0.62 (0.23–1.65)	.34	0.61 (0.23–1.64)	.33		
Parainfluenza	0.63 (0.27–1.48)	.289	0.62 (0.26–1.45)	.27		
Influenza A	1.17 (0.66–2.07)	.60	1.18 (0.66–2.10)	.57		
Influenza B	1.47 (0.67–3.25)	.34	1.45 (0.66–3.19)	.36		
SARS-CoV-2	**7.02 (4.32–11.43)**	**< .001**				
SARS-CoV-2 vaccine in exposed					1.52 (0.72–3.20)	.27

aOR, adjusted odds ratio, CI, confidence interval, VRI, viral respiratory infection, RSV, respiratory syncytial virus, hMPV, human metapneumovirus, SARS-CoV-2, severe acute respiratory syndrome coronavirus 2.

## Discussion

While shared rooms are already widely recognized to increase the risk of VRI, our study additionally observed increased risk associated with sharing a washroom between adjoining rooms. However, this finding was not observed with SARS-CoV-2 likely due to the smaller sample size and confounding by wider transmission on the unit.

A previous prospective study found a 2-fold higher odds of transmission of SARS-CoV-2 associated with shared washrooms of adjoining rooms.^6^ Similarly, in a mixed methods study of SARS-CoV-2 outbreaks in Australian aged care homes, institutions with single rooms only and no shared washrooms were associated with lower rates of resident cases, hospitalizations, and death.^7^ In contrast, linkage of SARS-CoV-2 surveillance data with a cross-section survey of the built environment of 134 long-term care homes in England found no association between the presence of washrooms shared between residents and SARS-CoV-2 transmission.^8^ Across these studies, the nature of shared washrooms was not consistently defined and non-SARS-CoV-2 VRI was not assessed.

Our study compared specific room designs across different VRIs due to the availability of multiplex RT-PCR for over a decade, while also adjusting for other covariates. Our findings support design standards for healthcare facilities in Canada that recommend single-resident rooms with a dedicated washroom.^9^ Guidelines from the United States do not directly address shared washrooms but recommend no more than 4 residents per room and 2 residents per toilet.^10^

For nursing homes with older infrastructure, shared washrooms between adjoining rooms are a reality yet have not historically been incorporated into VRI risk assessment. Our findings suggest that a resident who shares an adjoining washroom to a resident with VRI should be considered to be at increased risk with consideration given to implementing control measures. These may include use of a dedicated commode, maintaining a closed door or drawing curtains, heightened syndromic surveillance, and use of chemoprophylaxis for influenza as appropriate.^1,4,6^

Our study has important limitations. First, it was conducted at a single nursing home and the effects of a shared washroom could differ based on the room size and layout, and air balancing in different facilities. Second, we were unable to quantify additional sources of exposure including from healthcare workers, visitors and exposures amongst residents outside of the room such as at communal meals or activities; however, we did adjust for overall transmission dynamics on the unit during the time of exposure.

While further prospective studies are needed, measures recommended for exposed residents in shared rooms should be considered for those with shared washrooms between adjoining rooms.
